# Molecular Mechanisms of Zinc as a Pro-Antioxidant Mediator: Clinical Therapeutic Implications

**DOI:** 10.3390/antiox8060164

**Published:** 2019-06-06

**Authors:** Ananda S. Prasad, Bin Bao

**Affiliations:** Department of Oncology, School of Medicine, Wayne State University and Karmanos Cancer Center, Detroit, MI 48201, USA; baob@karmanos.org

**Keywords:** zinc, MT, NF-κB, A20, HNF-4α, ROS

## Abstract

The essentiality of zinc as a trace mineral in human health has been recognized for over five decades. Zinc deficiency, caused by diet, genetic defects, or diseases, can cause growth retardation, delayed sexual maturation, depressed immune response, and abnormal cognitive functions in humans. Zinc supplementation in zinc-deficient individuals can overcome or attenuate these abnormalities, suggesting zinc is an essential micro-nutrient in the body. A large number of in vitro and in vivo experimental studies indicate that zinc deficiency also causes apoptosis, cellular dysfunction, deoxyribonucleic acid (DNA) damage, and depressed immune response. Oxidative stress, due to the imbalance of reactive oxygen species (ROS) production and detoxification in the anti-oxidant defense system of the body, along with subsequent chronic inflammation, is believed to be associated with many chronic degenerative diseases such as diabetes, heart diseases, cancers, alcohol-related disease, macular degenerative disease, and neuro-pathogenesis. A large number of experimental studies including cell culture, animal, and human clinical studies have provided supportive evidence showing that zinc acts as an anti-oxidative stress agent by inhibition of oxidation of macro-molecules such as (DNA)/ribonucleic acid (RNA) and proteins as well as inhibition of inflammatory response, eventually resulting in the down-regulation of (ROS) production and the improvement of human health. In this article, we will discuss the molecular mechanisms of zinc as an anti-oxidative stress agent or mediator in the body. We will also discuss the applications of zinc supplementation as an anti-oxidative stress agent or mediator in human health and disease.

## 1. Introduction

The earliest clinical cases of human zinc deficiency, as manifested by dwarfism, sexual development delay in males, depressed immune response, and cognitive dysfunction, were observed by Dr. Prasad and his team in the Middle East in the 1960s [1]. There were no reports on zinc deficiency at that time. Iron treatment was initially given to those patients to correct the symptoms of anemia. However, iron supplementation only corrected the anemia and did not correct other symptoms [1]. Growth retardation, hypogonadism in males, and immune dysfunction were able to be corrected by zinc supplementation [1]. Zinc deficiency in the Middle East is caused by diets rich in organic phosphate compounds (phytates) which decrease the availability of zinc. Nutritional zinc deficiency is very common in developing countries. One recent study showed that there was zinc deficiency in around 43% of children aged 3–5 years in South Africa [2]. Another recent study showed that there was zinc deficiency in about 20% of children aged between 6 months and 12 years in Iran [3]. Moreover, conditioned zinc deficiency is also commonly found associated with some medical conditions such as chronic renal disease, diabetes, gastrointestinal diseases, sickle cell anemia, and cancers [4] Furthermore, zinc deficiency is widely prevalent in the elderly population worldwide, in part due to changes in lifestyle, diet, and health conditions. It has been estimated that 30–40% of elderly subjects have mild/marginal to modest zinc deficiency in the United States [5,6]. Zinc supplementation in elderly subjects improves their zinc and health status, for example through decreased incidence of infections [5,6,7].

Increased number of human studies shows that zinc deficiency is highly associated with increased levels of oxidative stress biomarkers such as lipid peroxidation products and DNA oxidation products in humans. Zinc supplementation suppresses or attenuates these adverse effects [5,6,7]. These findings suggest that zinc might have a protective role as a pro-antioxidant agent or mediator by the down-regulation of ROS production and accumulation. In the following paragraphs, we will summarize the biological functions of zinc as a ROS/oxidative suppressor or pro-antioxidant agent through several molecular mechanisms including anti-inflammatory effects and zinc-binding proteins such as nuclear factor κB (NF-κB), zinc containing transcription factor (A20), peroxisome proliferator-activated receptor (PPAR), tristetraprolin (TTP), hepatocyte nuclear factor-4α (HNF-4α), nuclear factor erythroid 2-related factor 2 (Nrf2), Kruppel-associated box domain (KRAB), and metallothionein (MT)/metal regulatory transcription factor 1 (MTF-1) in human health and diseases.

## 2. Physiological and Biological Functions of Zinc

Since human zinc deficiency was first reported by Dr. Prasad there has been no doubt as to the role of zinc as an essential micronutrient for human health [1]. Tremendous evidence shows that zinc plays very important roles in a variety of biological and physiological functions in humans [4,8]. For example, it is known that zinc participates in the activation of more than 300 enzymes that mediate the regulation of macromolecule biosynthesis in DNA, RNA, and proteins, as well as cell growth and proliferation and other types of metabolism [9]. The evidence also shows that zinc is required to stabilize the tertiary structures of more than 300 proteins (for example zinc finger proteins) and many transcription factors. These Cys2–His2 (C2H2)-enriched zinc finger domain proteins are reported to be involved in the control of gene expression of a wide variety of growth factors, steroid receptors, and immune response mediators by binding DNA, RNA, and proteins [10,11,12]. Inadequate zinc condition in the cells affects the structures of zinc-dependent proteins, subsequently leading to aberrations in protein activities, suggesting the important role of zinc as an essential micro-nutrient in humans.

## 3. Immunological Functions of Zinc

Immunological response is a very important biological process by which the body’s defense system recognizes and defends itself against the infection of microorganisms such as bacteria, viruses, and fungi as well as other substances that are foreign and harmful to the body. It is known that zinc acts as an immune mediator to maintain normal immune response against micro-organism infections in the body. A large number of clinical observations, as well as experimental and animal studies, have provided supportive evidence showing that zinc is involved in the regulation of several immune functions of T cells, B cells, and natural killer (NK) cells, specifically, cellular immunological function [13,14,15,16]. The evidence clearly suggests that zinc deficiency affects cellular immunological functions, for example resulting in decreased production of T helper 1 (Th1) cytokines and reduced DNA-binding activity of T-bet. T-bet is a major transcription factor for Th1 cytokine gene expression [17]. For example, zinc deficiency was found to decrease the expression and production of interleukin-2 (IL-2) and IL-2 receptors as well as interferon γ (IFN-γ) cytokines in HUT-78 cells (a type of Th_0_ malignant lymphomatoid cell), as compared to zinc-sufficient cells [17,18,19]. Dysfunction of cellular immune response caused by zinc deficiency in humans causes frequent severe fungal, bacterial, and viral infections, thymic atrophy, anergy, decreased proliferative response of serum lymphocytes to mitogens (a selective decreased population of T helper cells), and decreased activity of thymulin, a thymic hormone that induces the differentiation of T-cells and enhances the functions of the different T-cell subsets. Zinc supplementation reverses all of these manifestations in the body [20,21]. These findings clearly suggest that zinc could be an important immune responsive mediator.

## 4. ROS, Oxidative Stress, and Inflammatory Response

Chemically, in the presence of a free electron (e), the univalent reduction of oxygen generates superoxide (•O_2_^−^), hydrogen peroxide (H_2_O_2_), and hydroxyl radicals (•OH), all of which are reactive oxygen species (ROS). Superoxide has an unpaired electron, which imparts higher reactivity and renders it very unstable and short-lived [22,23,24,25]. ROS are usually produced continuously in vivo under aerobic conditions. The production of ROS and its elimination by the anti-oxidant defense system in cells is a highly modulated process for maintaining normal physiological function in the body. In eukaryotic cells, the mitochondrial respiratory chain, microsomal cytochrome p450 enzymes, flavoprotein oxidases, and peroxisomal fatty acid metabolism are the most significant intracellular sources of ROS [26,27,28,29]. The nicotinamide adenine dinucleotide phosphate (NADPH) oxidases are a group of plasma membrane-associated enzymes which catalyze the production of superoxide •O_2_^−^ from oxygen by using NADPH as the electron donor [30]. Broadly speaking, ROS cover reactive nitrogen species (RNS) including radicals such as nitric oxide (NO•) and nitric dioxide (NO_2_), as well as non-radicals such as nitrous acid (HNO_2_) and dinitrogen tetroxide (N_2_O_4_). ROS, including reactive nitrogen species (RNS), are considered as second messengers for important molecular signaling transductions [31]. Similar to second messengers, the production of ROS is highly triggered by extra-cellular stimuli such as hypoxia, growth factors, and inflammatory cytokines. Reduction-oxygenation (redox) signaling involves at least one reaction with oxidation of a signaling molecule by ROS, which is reversible [32].

The homeostasis of ROS has been implicated in a variety of biological responses from transcriptional activation to cell proliferation. Under pathological conditions, a disequilibrium between ROS production and elimination by the antioxidant defense system results in increased bio-availability of ROS, leading to a state of oxidative stress, a deleterious process [29,33]. The pathogenic outcomes of oxidative stress involve oxidative damage of cells or tissues [23], a major cause of DNA damage. The failure of the repair of this ROS-induced DNA damage by the DNA repair system in the body leads to genomic instability or mutations, subsequently contributing to the development of many chronic degenerative diseases such as cancers and diabetes.

The inflammatory response is a complex and critical immunological process resulting from the host defense mechanisms against trauma, micro-organism infection, and other adverse environmental stresses or complex combinations of many biological insults by the involvement of several different immune cells such as lymphocytes, macrophages, NK cells, dendritic cells, and other cells, as well as many different cytokines/molecules [34,35,36,37,38]. Such inflammatory processes, especially sustained chronic conditions of inflammation, along with inflammation-induced oxidative stress from dead or injured cells, could lead to irreversible cellular or tissue damage with the passage of time, which further contributes to the development of chronic degenerative diseases.

As discussed in Section 6.1, ROS are a potent stimulus for the activation of NF-κB, a major transcription factor that regulates the expression of many inflammatory genes. The over-production of ROS, namely oxidative stress, results in the chronic, sustained constitutive activation of the NF-κB signaling pathway, eventually leading to the up-regulation of inflammatory cytokines. Thus, oxidative stress, along with sustained, chronic inflammatory responses acts as a deleterious contributor for the development of many chronic degenerative diseases such as atherosclerosis, rheumatoid arthritis, and diabetes mellitus in humans, mediated through the oxidation of macro-molecules such as DNA, lipids, and proteins and ROS-induced inflammatory response processes [39,40,41].

## 5. Zinc as an Anti-Oxidative Stress and Anti-Inflammatory Agent 

From a chemical perspective, zinc is a redox-inert ion which cannot oxidize nor reduce other substances in the body, and is not a direct anti-oxidant agent itself [42]. Zinc can bind to the sulfur (thiolate) donor of cysteine to form zinc thiolate, which turns zinc redox-active. Oxidants can interact with thiolate and release zinc in a free state. Such oxidative releases of zinc from thiolate donors of cysteine residues generate a zinc signal that triggers an anti-oxidant response against ROS/oxidative stress. Physiological or adequate levels of zinc have a pro-antioxidant effect or protective effect against ROS/oxidative stress in biology. Extreme zinc levels such as severe deficiencies and toxicological or excess levels of zinc in the body exert pro-oxidant effects, causing oxidative stress, as reviewed recently [42,43]. Zinc has been proposed to act as a pro-antioxidant agent or a co-factor by three mechanisms: (1) protection of free sulfhydryl group in proteins; (2) outcompeting redox-active metals; and (3) specific induction of antioxidant system response [42,43]. A large number of early experimental studies revealed that physiological or adequate levels of zinc exert an anti-oxidant effect in a site-specific pattern in the body by the regulation of the highly cysteine-rich and heavy metal-binding protein metallothionein (MT) [42,44]. The evidence shows that zinc directly increases the expression and activity of MT, which is also known as a zinc binding protein and is highly rich in cysteine, thus being an excellent scavenger of ˙OH from the zinc thiolate of cysteine residues [45,46], as discussed further in Section 6.8.

A large number of clinical and experimental studies have indicated that zinc deficiency is highly associated with increased levels of oxidative stress biomarkers, DNA damage, and inflammatory cytokines/molecules in humans. Zinc supplementation reverses these adverse effects. For example, it has been reported that zinc-deficient human lung fibroblast cells in cultured conditions not only increase oxidative stress and DNA damage but also decrease DNA repair capacity [47,48]. The evidence shows that zinc deficiency decreased the DNA binding activities of p53, NF-κB, and activator protein 1 (AP-1), also causing an increase in the generation of oxidative stress and the expression of DNA repair proteins [47,49]. It is noted that after tumor nectosis factor-α (TNF-α) treatment, zinc-deficient porcine vascular endothelial cells showed increased levels of oxidative stress and inflammatory cytokine IL-6 as well as activation of NF-κB and AP-1 [50]. One animal study showed that zinc-deficient rats had higher concentrations of thiobarbituric acid-reactive substances (TBARS, well-known lipid peroxidation biomarkers) in the liver, brain, and testes [51]. These findings clearly suggest that zinc might have an important role in the maintaining ROS homeostasis in the body.

Our early cell culture models and clinical trials from normal healthy individuals, elderly subjects, and sickle cell anemia subjects demonstrate that zinc decreased nitric oxide (NO) production, lipid peroxidation products, DNA oxidation products, inflammatory cytokines/molecules, and inducible NO synthase (iNOS) in human subjects, and also inhibited the activations of TNF-α-, oxidized low density lipoprotein (LDL-), or lipopolysaccharide (LPS)-induced NF-κB DNA binding in human promyelocytic leukemia cells (HL-60), human monocytic leukemia cells (THP-1), human aortic endothelial cells (HAECs), or human isolated peripheral blood mononuclear cells (PMNC) [6,7,12]. Similar results have been reported by other investigators [52,53,54,55,56]. These findings strongly suggest that zinc reduces oxidative stress and ROS-mediated inflammatory responses, and that zinc acts as a potent agent by inhibition of ROS production and inflammation.

## 6. Molecular Mechanisms of Zinc as an Anti-Oxidative Stress Agent

A large number of experimental studied have provided valuable evidence to support zinc as an anti-oxidative stress agent by targeting several zinc-associated or zinc finger proteins such as NF-κB, A20, TTP, PPARs, Nrf2, HNF-4α, KRAB-containing proteins, and MT/MTF-1 in the body, as discussed in Section 6.1, Section 6.2, Section 6.3, Section 6.4, Section 6.5, Section 6.6, Section 6.7 and Section 6.8.

### 6.1. NF-κB 

Nuclear factor κB (NF-κB) is a widely studied major transcription factor which regulates many different gene expressions involved in immune, inflammatory response, and other biological processes [57]. Alterations in the NF-κB signaling pathway activity are strongly related to the development and progression of age-related chronic diseases, for example, atherosclerosis, rheumatoid arthritis, diabetes, malignant tumors, and other diseases. NF-κB is highly activated by different intrinsic and extrinsic stimuli such as inflammatory cytokines/molecules, ROS, protein kinase C (PKC) activators, UV light/ionizing radiation, and other stresses [58,59]. NF-κB activation and its signaling pathway regulate the expressions of many different inflammatory cytokines/molecules, transforming growth factor, adhesion molecules (such as intracellular adhesion molecule (ICAM-1), vascular cell adhesion molecule-1 (VCAM-1), and E-selectin), receptors (IL-2 receptor-α), oxidant molecules/enzymes (inducible NO synthase and inducible cyclooxygenases), and other immune molecules/mediators, leading to the mediation of several immune responses including innate and adaptive immune response, stress response, and cell survival and proliferation in the body. Inflammatory cytokines such as TNF-α and interleukin 1β (IL-1β) not only activate the NF-κB signaling pathway but also are induced by the activation of the NF-κB signaling pathway [58,59], which results in a positive feedback-loop of amplification with sustained chronic activation of the NF-κB signaling pathway in many different cells such as immune cells and cancer cells, eventually leading to pathological processes. NF-κB proteins consisting of five different isoforms such as p65 (RelA), RelB, c-Rel, p105/p50 (NF-κB1), and p100/p52 (NF-κB2), rather than acting alone, can usually co-operate with other transcription factor proteins such as activator protein 1 (AP-1), specific protein 1 (SP-1), and CCAAT enhancer binding protein (C/EBP) (NF-κB–bZip interactions), to enhance the expressions of many different genes with a variety of molecular and cellular functions in a cell type-dependent pattern [60,61].

If no stimulation or exposure to stress occur, NF-κB proteins in the cytoplasm are not activated by binding to inhibitory proteins of NF-κB (IκB) such as IκB-α, IκB-β, and IκB-γ subunits [58,59,60]. The activation of NF-κB is mediated through its dissociation from the binding of IκB proteins to NF-κB proteins. Thus, cytosolic NF-κB proteins become an active form for translocation into the nucleus only when IκB proteins are released from the NF-κB proteins in the cytoplasm. Many stimuli such as cytokines, PKC activation, oxidants, UV light, and radiation can induce the activation of NF-κB via phosphorylation of IκB proteins mediated through the action of IκB kinases (IKK), which finally results in ubiquitination and subsequent degradation of IκB proteins by the proteolytic enzyme-enriched proteasome complex in the cells [58,59,60]. Dissociations of IκB proteins from NF-κB proteins cause their rapid nuclear translocation for the activation of NF-κB targeted genes. Aberrations of NF-κB activation cause alternations in the expression of many different NF-κB-targeted genes, which contribute to the regulation of many different molecular and cellular functions such as cell growth, proliferation, differentiation, apoptosis, and immune response in the body.

The data from several early experimental studies have provided solid evidence to support the concept that zinc plays a critical role in the modulation of NF-κB signaling pathway activity, and that the regulation of NF-κB activation by zinc appears to be cell lineage-dependent [62,63]. For example, the evidence from early in vitro studies has shown that zinc is required for the activity of NF-κB and its protein-DNA binding in either purified NF-κB proteins, recombinant NF-κB p50, or Th_0_ cell line (HUT-78)-derived nuclear protein extracts, although one early report has revealed that there is no zinc-binding site in the structures of NF-κB proteins [18,64,65]. One early animal study showed that rats undergoing a short period (14 days) of diet-induced zinc deficiency had lower levels of NF-κB DNA binding activity in testes, along with increased levels of oxidative stress, suggesting that the reduction of NF-κB binding reflects an early response to zinc deficiency-induced oxidative stress [66]. However, many other experimental studies in cell culture models have revealed that zinc can suppress LPS-, ROS-, or TNF-α-induced NF-κB activation in endothelial cells, pancreatic cancer cells, embryonic fibroblast cells, and PMNCs, consistent with a decrease in the expression/production of inflammatory cytokines and oxidative stress [7,47,48,49,67,68,69,70,71,72,73]. These findings clearly suggest that the regulation of the NF-κB signaling pathway activity by zinc is cell type-specific. More studies on the specificity of zinc on NF-κB activation in different types of cells are required for further investigation.

### 6.2. A20

A20 has been widely recognized as an endogenous inhibitor of NF-κB activation, and is different from IκB protein. A20 (also known as TNF-α-induced protein 3, TNFAIP3) is a cytoplasmic zinc finger transactivating factor that plays an important role in the inhibitory regulation of inflammatory response via the inhibition of IL-1β- and TNF-α-induced NF-κB signaling pathway activity [74,75,76]. The expression of A20 is mainly induced for the inhibitory regulation of NF-κB signaling pathway activity in many different types of cells in response to a number of stimuli such as TNF-α, IL-1β, LPS, phorbol myristate acetate (PMA), and ROS as well as other stimuli [76]. A20 was originally identified to protect cells from TNF-α-induced cytotoxicity by the suppression of the activity of the NF-κB signaling pathway, which leads to a decrease in TNF-α and IL-1β signaling pathways in endothelial cells and other cells [76]. It has been shown that the genetic A20 null (A20^−/−^) mice can develop severe inflammation and cachexia, and are highly sensitive to both LPS and TNF treatments, with death in the early stage after birth [77]. A20 knockout (A20-deficient) cells cannot suppress TNF-induced NF-κB responses. Early evidence also revealed that A20 inhibited the TNF-α- and IL-1β-activated NF-κB signaling pathway by mediating TNF-receptor associated factor (TRAF) pathways in endothelial cells [74,75,78]. More evidence shows that A20 contains two ubiquitin-editing domains for de-ubiquitinating proteins to inhibit NF-κB signaling pathway activity by inhibition of the ubiquitination of IκB proteins [79,80]. All of these findings clearly support the concept of A20 as an endogenous anti-inflammatory molecule by the inhibition of NF-κB activation via TRAF pathways. The inhibitory effects of zinc on NF-κB-mediated inflammation and oxidative stress have been considered to be associated with the expression of A20 protein in non-T cells [70]. Our previous experimental studies have shown that zinc sufficiency increases A20 expression and A20–TRAF1 complex binding, and decreases the generation of inflammatory cytokines and oxidative stress in non-T lymphocyte cells such as (HL-60) human promyelocytic leukemia cell line, (TPH-1) the human monocytic leukemia cell line, and (HUVEC) the human umbilical vein endothelial cells, as compared to zinc deficiency in those cells [7,69,70]. The under-expression of A20 by its anti-sense mRNA increases the generation of inflammatory cytokines such as TNF-α and IL-1β in zinc-sufficient cells [70]. These findings clearly suggest that zinc suppresses the generation of inflammatory cytokines and ROS via the regulation of A20 signaling pathway, as a result of deregulation of NF-κB activity. One recent in vitro study showed that zinc could suppress LPS-induced NF-κB activation by induction of A20 expression in RAW 264.7 macrophage cells [81]. More evidence from a recent animal study revealed that zinc treatment could inhibit NF-κB activation via the induction of A20 expression in rats [81]. Therefore, zinc may act as an anti-inflammatory and anti-oxidative stress agent, partially being associated with the modulation of A20 expression.

### 6.3. TTP 

Tristetraprolin (TTP; also known as Nup475, TIS11, or Zfp36), another known zinc finger protein, is one of three family members of CCCH tandem zinc finger proteins in mammals, characteristic of two CCCH zinc finger domains and three tetraprolin motifs [82,83,84]. TTP is known to bind class II AU-rich elements of mRNAs, specifically encoding TNF-α and granulocyte/macrophage colony-stimulating factor. Such RNA–protein binding results in the destabilization of these mRNAs by removal of poly A tail of these mRNAs, decreased translation, and depressed secretion of these cytokines/molecules [82,83,84]. TNF-α is a strong inflammatory cytokine which can induce/activate the NF-κB signaling pathway [83,85]. Early animal studies indicate that TTP deficiency causes chronic inflammatory syndromes which are similar to human rheumatoid arthritis and gastrointestinal inflammatory syndromes by the increased stabilization of TNF-α mRNA, and consequent over-production of this cytokine in mice [86,87,88,89,90]. Recently, one animal study showed that TTP-null (TTP-deficient) mice had increased levels of reactive oxygen and nitrogen species, along with the activation of NADPH oxidase 2 (NOX2), which contributes to chronic inflammatory syndromes in the TNF-α-independent manner [89], suggesting a potential role of TTP in the regulation of ROS homeostasis. The early experimental study in several types of cultured cells and mice indicates that a zinc-sufficient condition (100 μM) up-regulates the gene expression of TTP [91]. These findings suggest that TTP may have a protective role against oxidative stress mediated through the inhibition of TNF-α by zinc.

### 6.4. PPAR

Peroxisome proliferator-activated receptors (PPARs) α and γ in nuclear receptors have widely been known as potent mediators for lipoprotein metabolism, inflammation, and glucose homeostasis in the body for decades and shown to play an important protective role in the development and progression of several age-related degenerative diseases such as atherosclerosis, diabetes, and cancer [92,93,94]. PPAR-α and γ are extensively expressed in many different tissues such as the liver and pancreas, and are abundantly present in the vascular vessels where they may exert anti-inflammatory effects [95,96]. Increasing evidence has shown that PPAR proteins have anti-inflammatory properties [97,98,99,100]. Clinically, several PPAR agonists have been used for the treatment of cardiovascular disease (CVD) or patients with high risk for CVD such as those with diabetes [101], in part associated with its inhibition of inflammatory response. Although the mechanisms of PPAR-α and γ agonist drugs against these degenerative diseases have not been fully elucidated, increasing evidence shows that one of the mechanisms may be due to its anti-inflammatory and anti-oxidant effects through the inhibition of oxidant-sensitive NF-κB activation via a negative cross-talk at the nuclear DNA binding level [102]. The evidence also reveals that the activation of PPAR-α and γ leading to the inhibition of inflammatory cytokines/molecules in cells is believed to be zinc-dependent [103].

One early experimental study revealed that after the zinc depletion induced by TPEN (*N*,*N*,*N*′*N*′-tetrakis (2-pyridylmethyl) ethylenediamine), a zinc-specific chelator, in porcine pulmonary arterial endothelial cells, the PPAR-α or PPAR-γ agonist-pre-treated cells lost the potency to inhibit TNF-α-induced NF-κB and AP-1 DNA binding activities. However, when zinc was added back into the zinc-depleted cells, PPAR agonists significantly inhibited these inflammatory parameters [104]. Another experimental study revealed that zinc deficiency induced inflammatory cytokines/molecules associated with NF-κB and PPAR signaling pathways [94]. In our previous experimental study, human aortic endothelial cells (HAECs) cultured in zinc-deficient conditions (1 μM of zinc) resulted in a decrease in the expression of PPAR-α protein after oxidized LDL stimulation, as compared to those cells cultured in zinc-sufficient conditions (15 μM of zinc) [7], suggesting that zinc increases the expression of PPAR-α, which may contribute to the inhibition of NF-κB-mediated inflammatory cytokines/molecules. Therefore, the protective role of zinc as an anti-inflammatory and anti-oxidative stress agent may be partially associated with zinc-dependent PPAR signaling pathways.

### 6.5. Nrf2

Nuclear factor erythroid 2-related factor 2 (Nrf2), another known zinc finger protein, is a family member of Cap’n’Collar/basic leucine zipper (CNC-bZIP) proteins, and is known as a critical transcription factor that regulates the gene expressions of anti-oxidant proteins and enzymes such as glutathione (GSH) and superoxide dismutase (SOD). It also detoxifies enzymes such as glutathione S-transferase-1 (GSTA1) and heme oxygenase-1 (HO-1), by binding to anti-oxidant responsive elements (ARE) in the DNA promoter regions of these target genes [105,106]. It is known that these anti-oxidant molecules/enzymes and phase II detoxifying enzymes have a protective role in anti-oxidant defense system against electrophilic stressors and oxidative stress in cells [106]. The data from a large number of experiments have provided clear evidence supporting the concept that Nrf2 plays an important cytoprotective role in the regulation of oxidative stress-induced cellular damage in the body [107,108]. It has been revealed that in aged rats there is an approximately 50% reduction of Nrf2 activity and ARE binding activity in the liver, associated with a low level of HO-1 [106]. Therefore, Nrf2 has been considered as an anti-oxidant mediator in the anti-oxidant defense system through the regulation of these anti-oxidant proteins and enzymes in the body. Deregulation of Nrf2 expression has been shown to be associated with the development of age-related degenerative diseases including cancers and diabetes [107,109,110,111].

The evidence from several recent experimental studies suggests that zinc may have an important role in the regulation of Nrf2 expression. One experimental study revealed that zinc depletion induced by administration of TPEN (a known zinc-specific chelator) to mice increased oxidative stress and decreased Nrf2 activity [112]. A high dose of zinc increased non-protein thiol levels, HO-1 expression, and nuclear Nrf2 protein in human colon cancer HCT-116 cells [113]. It has also been documented that zinc can prevent H_2_O_2_-induced endothelial cell damage via Nrf2-dependent stimulation of glutathione biosynthesis, and zinc depletion has a reverse effect on ROS and Nrf2 [114]. Moreover, dietary zinc supplementation can restore bacterial clearance in the lung, along with an increase in nuclear Nrf2 binding activity in alveolar macrophages, and decrease oxidative stress in alcohol-fed rats [108]. These findings clearly suggest that zinc up-regulates Nrf2 activity and inhibits the generation of oxidative stress. Thus, the regulation of Nrf2 by zinc may be one of the molecular mechanisms of zinc as an anti-oxidative stress agent in the body. More studies are required to investigate the protective role of Nrf2 in inflammation and oxidative stress by zinc.

### 6.6. HNF-4α

Hepatocyte nuclear factor-4α (HNF-4α) is a zinc finger transcription factor and is mostly expressed in the liver in the body. This zinc finger transcription factor regulates the expression of a large number of genes which are involved in several aspects of hepatocyte functions such as cell proliferation and apoptosis [115,116]. Increasing evidence has shown that HNF-4α has an important protective role against alcohol-induced and TNF-α-induced cellular damage in the liver [117,118,119]. Moreover, one recent animal study indicates that HNF-4α null (HNF-4α-deficient) mice significantly had significantly impaired expression of transferrin, resulting in hypoferremia, a state of abnormal metabolism of Fe, which may cause oxidative stress in liver cells [120]. These findings suggest that HNF-4α regulates the homeostasis of Fe metabolism, which is associated with the ROS signaling pathway.

Several experimental studies show that zinc supplementation increased the expression of HNF-4α in liver and has a protective role in alcohol-induced liver disease in animals. Specifically, one animal study demonstrated that zinc supplementation reversed alcoholic liver disease by the re-activation of HNF-4α and PPAR-α in mice. Zinc depletion significantly down-regulated HNF-4α and PPRA-α downstream target proteins in liver cells [118]. Other experimental studies indicate that zinc deficiency decreased cell proliferation and related proteins such as hepatocyte growth factor (HGF), insulin like growth factor I (IGF), IGF binding protein 1 (IGFBP-1), MT, and cyclin D1, along with HNF-4α protein. The functional loss of HNF-4α by its small interfering ribonucleic acid (siRNA) has similar effect to zinc deficiency [118,121]. These findings suggest zinc-mediated HNF-4α might have an important protective role against oxidative stress by several molecular signaling pathways.

### 6.7. KRAB Proteins

Kruppel-associated box domain (KRAB) proteins are a large family of zinc finger transcription factors, which were discovered in 1991 [122]. They are also called as KRAB-containing zinc finger proteins. There are nearly 800 different KRAB-containing proteins in tetrapod vertebrates. However, only one third (around 300) of KRAB-containing proteins have been found in the human genome, although they represent the most of the zinc finger proteins in humans. KRAB-containing proteins are characterized by the presence of a DNA-binding domain consisting of 4–30 zinc finger motifs and a KRAB domain, which is located near the amino terminal of the proteins [123].

KRAB domain has been identified to act as a transcription repressor domain by binding to co-repressor proteins, whereas the C2H2 zinc finger motifs of these proteins binds to DNA elements of the target genes. The functions of these KRAB-containing proteins currently proposed are the transcriptional repression of RNA polymerase I, II, and III promoters as well as binding and splicing of RNA. The members of the KRAB-containing protein family are considered to involve in the maintenance of the nucleus, cell differentiation, growth, proliferation, cell apoptosis, and neoplastic transformation [122].

Recent experimental studies have demonstrated that several KRAB-containing proteins such as zinc finger protein 552 (ZNF552) (407 amino acids at length with one KRAB domain at the amino terminal and seven C2H2 zinc finger motifs at the carboxyl terminal), ZNF328 (792 amino acids at length with a N-terminal KRAB domain and classic C-terminal C2H2 zinc finger motifs), and ZNF649 (505 amino acids of protein with N-terminal KRAB domain and C-terminal C2H2 zinc finger motifs) significantly inhibited serum response element (SRE) and AP-1 activities in COS-7 kidney fibroblast cells [124,125,126]. AP-1 is one key transcription factor for redox signaling pathway. Zinc deficiency has been found to induce ROS and AP-1 in 3T3 mouse embryonic fibroblast cells [73]. Thus, the regulation of KRAB-containing protein homeostasis by zinc would regulate AP-1-mediated redox signaling pathway.

### 6.8. MT and MTF-1

Metallothionein (MT) is another well-known zinc binding protein, which has been widely studied for several decades, and is a group of metal binding proteins characteristic of low molecular weight (ranging from 500 to 1400 Da) with a single peptide chain [45]. One third of its amino acids are cysteines distributed in two domains α and β clusters, contributing to high binding affinity to seven ions of divalent metals such as zinc, iron (Fe), copper (Cu), selenium (Se), and cadmium (Cd) through the thiol group (SH) in cysteine residues in the body [45]. To date, four isoforms of MT proteins, namely, MT-I, II, III, and IV, have been identified. MT-I and MT-II are predominantly expressed in many different tissues such as liver, kidney, intestine, and brain in humans [127].

The key function of MT is to mediate the homeostasis, storage, and transport of essential trace elements such as zinc and Cu as well as detoxification in the body [44,128,129]. Other metal elements including heavy metals such as Cd in the body can also bind to MT through its thiol group. Due to the presence of multiple thiol groups of its cysteine residues, MT has been considered as an anti-oxidant molecule in the cellular defense antioxidant system in the body by acting as a free radical scavenger [44,127,130,131,132]. The evidence shows MT has an anti-inflammatory effect by the modulation of the expression of several pro- and anti-inflammatory cytokines [127,133,134], contributing its anti-oxidant effect in the body. Many animal studies show that MT clearly have a protective role against oxidative stress-induced damage in many different tissues as reviewed recently [127]. The regulation of MT gene expression is zinc-sensitive. A variety of stimuli such as metal exposure, ROS/oxidative stress, and steroids also induce the gene expression of MT. Many experimental studies show that zinc supplementation induces MT gene expression [135,136]. One animal study shows that zinc supplementation induced the concentration of tissue MT and antioxidant enzymes and decreased the levels of tissue Cu and Fe as well as Cu- and Fe-associated free radicals in rats [137], suggesting that the induction of MT synthesis by zinc contributes to the reduction of free radicals generated by Cu and Fe.

Metal response element (MRE) binding transcription factor 1 (MTF-1) was identified as a zinc finger protein in the 1990s [138]. This protein has six zinc finger motifs in the Cys2–His2 family. Increasing evidence shows that MTF-1 is involved in zinc homeostasis and cellular responses to heavy metals, hypoxia, ROS/oxidative stress, and ionizing radiation by the regulation of metal-dependent (especially zinc-dependent) induction of targeted genes containing the MRE DNA sequence in the promoter region [139]. Upon the binding of zinc to its zinc finger motifs due to the change in intracellular free zinc concentration, MTF-1 induces the transcription activation by its binding to MRE site in the promoter region of targeted genes encoding MT1, MT2, zinc transporter1 (Znt1, a zinc efflux transporter protein), and the glutamate–cysteine ligase heavy chain (an oxidative stress-related protein) [136,139,140,141]. This is why MTF-1 is known as a zinc sensor in the cells. The exact molecular mechanism and function of MTF-1 activation is not fully understood. Besides high sensitivity to zinc, ROS, specifically H_2_O_2_, can induce MTF-1 activation, resulting in the increased expression of MT proteins [44,139]. Experimental studies show that zinc supplementation increased MTF-1 DNA binding activity and MT-1 in vitro and in vivo [142,143,144]. This suggests that zinc has protective effect against ROS/oxidative stress, partially via the activation of the MTF-1/MT signal pathway. However, further studies are required to elucidate how zinc act as a pro-antioxidant agent or ROS/oxidative stress suppressor, in part by modulation of the MTF-1/MT signal pathway.

## 7. Applications of Zinc Supplement as a Pro-Antioxidant Mediator in Human Health

Zinc has been widely considered not only as an immune mediator, but also as a pro-antioxidant mediator in human health and disease. Therefore, the supplementation of zinc as a pro-antioxidant mediator against oxidative stress would have beneficial effects in different groups, for example the elderly and patients with age-related macular degenerative disease, sickle cell anemia, diabetes mellitus, (especially type 2), and alcohol-related liver disease, as discussed in Section 7.1, Section 7.2, Section 7.3, Section 7.4, Section 7.5 and Section 7.6.

### 7.1. Normal Adult Subjects

We at first reported the supplementation of zinc as a pro-antioxidant mediator in human adult subjects [69]. In our earlier human subject study in healthy adults, we found that oral zinc supplementation (45 mg zinc as gluconate per day for eight weeks) in normal healthy volunteers resulted in the significant decrease in the blood lipid peroxidation products malonyl dialdehyde (MDA) and 4-hydroxyalkenals (HAE), and DNA oxidation products (8-OHdG), compared to the placebo group of normal healthy subjects. Zinc supplementation attenuated TNF-α-induced NF-κB activation from isolated mononuclear cells (MNCs), compared to the MNCs isolated from the placebo group of healthy subjects who did not receive zinc supplementation. Zinc supplementation also decreased the mRNA levels of TNF-α and IL-1β in LPS-stimulated MNCs [69]. In our other clinical trial studies, we found that zinc treatment can significantly decreased the duration and severity of common colds in association with a decrease in inflammatory cytokines/molecules in normal healthy subjects [145,146]. These findings suggest that zinc may act as an anti-oxidative stress and anti-inflammatory mediator in human health.

### 7.2. Elderly Subjects

It is clear that aging is associated with oxidative stress, leading to macro-molecule damage and consequently chronic inflammation in the body. Due to changes in the dietary habits, lifestyle, and health conditions, zinc deficiency is very common in the elderly population. It has been estimated that 30–40% of elderly subjects have mild to moderate zinc deficiency in the United States [5]. A large number of experimental studies support the fact that zinc plays very important roles in the human health, especially in elderly subjects [147,148,149,150]. Zinc supplementation for the elderly would provide beneficial health effects [150,151]. For example, in our earlier studies, we found that so-called healthy elderly subjects had increased levels of blood lipid peroxidation products (MDA and HAE) and DNA oxidation intermittent products (8-OHdG) as well as inflammatory cytokines/molecules as compared to young healthy subjects [6,7,152]. We administered oral zinc supplementation (45 mg zinc for 6 months) to healthy elderly subjects. After 6 months of follow-up, zinc supplementation resulted in increased blood zinc levels and anti-oxidant power as well as ex vivo production of interleukin-2 (IL-2) and interferon γ (IFN-γ) and decreased blood levels of inflammatory cytokines/molecules such as high-sensitivity C-reactive protein (hsCRP), IL-6, macrophage chemoattractant-1 (MCP-1), vascular cell adhesion molecule-1 (VCAM-1), intracellular adhesion molecule (ICAM), and secretary phospholipase A2 [6,7,152], as well as ROS biomarkers such as MDA and HAE, as compared to the placebo group of healthy elderly subjects [6,7,152]. Zinc supplementation also decreased the incidence of infection in these elderly subjects [6,7,152]. Another clinical trial study in elderly subjects also provides solid evidence showing that zinc supplementation enhanced anti-oxidant defense system of peripheral blood lymphocytes by the up-regulation of activities of protein maintenance systems responsible for the elimination of oxidized protein in elderly subjects [153]. All of these findings confirmed that zinc may have anti-oxidant and anti-inflammatory effects, which could be useful for maintaining normal health conditions in elderly population who are vulnerable to infections and many other chronic degenerative diseases.

### 7.3. Age-Related Macular Degeneration Disease

Age-related macular degeneration (AMD) disease is one of the leading causes of permanent, irreversible, and central vision loss (blindness) in individuals aged over 50 worldwide [154]. It has been estimated that approximately 2 million individuals in the United States and 50 million elderly persons worldwide suffer from AMD [154]. AMD is a complicated chronic neurodegenerative and progressive disease. Its etiology is complicated and is not fully understood. Multiple factors are involved in the pathogenesis of AMD. It has been known that aging and genetics play a critical role in the development of AMD. However, other environmental factors such as cigarette smoking, heart disease, hypertension, dyslipidemia, obesity/diabetes, and phototoxic exposure that are associated with oxidative stress also play an important role in the development of AMD [155].

It has been documented that excessive production of ROS with oxidative stress play a key role in the pathogenesis of AMD [156,157,158]. The increased levels of ROS and suppressed cellular antioxidant defense system can result in oxidative stress and the consequent damage to photoreceptors, retinal pigment epithelial cells, and choriocapillaris, as reviewed recently [159]. Thus, the supplementation of dietary anti-oxidant agents or mediators to elderly subjects would provide a protective effect on AMD.

One large multi-center study supported by the national Institute of Health/the National Eye Institute, namely the Age-Related Eye Disease Study (AREDS) in the United States, enrolled 4700 participants aged 55 years or older, reporting that the supplementation of antioxidants including zinc provided a protective role in the development of AMD. Specifically, a long period (more than five years) of either the supplementation of 80 mg zinc as zinc oxide and 2 mg copper as cupric oxide or the supplementation of antioxidants (vitamin C, vitamin E, and beta-carotene) plus zinc significantly reduced the risk of developing advanced AMD [160]. A high dose (greater than 45 mg) of zinc supplementation or intake may impact copper absorption and cause copper deficiency due to its competition to copper binding protein or transporter in the body. Copper supplementation was administered to prevent copper deficiency caused by a high dose of zinc intake. The report showed that zinc alone decreased the odds of developing advanced dry type of neovascular form of AMD and prevented blindness in AMD subjects. It was also reported that only zinc showed a 27% decrease in mortality of AMD subjects, due to decreased incidence of adverse cardiovascular events [161,162]. Another report from the Rotterdam Study showed that high dietary intake of zinc reduced the hazard ratio of early AMD from 2.25 to 1.27 [163]. A recent systematically report supports that high level of zinc intake has a beneficial effect on progression of dry-type AMD in the elderly subjects [164], suggesting that anti-oxidants or its mediators including zinc have a beneficial effect on the development of AMD in elderly subjects.

### 7.4. Sickle Cell Anemia

Sickle cell anemia (SCD) is an inherited disease, a condition in which there are not enough healthy red blood cells to carry adequate oxygen throughout the body. The disease is caused by a mutation in the gene encoding iron-rich hemoglobin. Hemoglobin allows red blood cells to carry oxygen from the lungs to all parts of the body. In sickle cell anemia, abnormal hemoglobin causes red blood cells to become rigid, sticky, and misshapen. In sickle cell disease, a single amino acid substitution in β-chain at the sixth position takes place (Val replaces Glu). The sickle cell gene is passed from generation to generation in a pattern of inheritance called autosomal recessive inheritance. This disease is commonly seen in the African American population in the United States.

It has been documented that zinc deficiency is very common in adult SCD subjects [165,166,167,168,169,170]. We previously reported that approximately 60–70% of adult SCD subjects had zinc deficiency based on the plasma zinc level in our medical center [165,166,167,168,169]. These SCD patients with the lower levels of zinc had growth retardation, hypogonadism in male subjects, abnormal dark adaptation, hyperammonemia, and cell-mediated immunological abnormality. The clinical trials showed that zinc supplementation to those SCD patients who had zinc deficiency increased growth and gonadal development in male subjects, improved dark adaptation, decreased plasma ammonia levels, corrected anergy, and increased cell-mediated immunological function [165,166,167,168,169,170,171].

Increasing evidence shows that SCD patients also have an aberration of ROS homeostasis in body, which can worsen the progress of SCD. Thus, zinc supplementation would improve the status of ROS in SCD patients, attenuating the development and progression of SCD. Our clinical trial demonstrated that zinc supplementation (25 mg zinc for 3 months) decreased the incidence of infection, compared to the placebo subjects who did not receive zinc supplementation in SCD patients. After 3 months of follow-up, red blood cells, hemoglobin, hematocrit, plasma zinc, and anti-oxidant power significantly increased; plasma levels of nitrite and nitrate, lipid peroxidation products measured by MDA and HAE, and DNA oxidation intermediate products (8-OHdG), soluble VCAM-1 decreased in zinc-supplemented SCD patients, as compared to the placebo group. Three months of zinc supplementation to those SCD patients also showed significant decreases in LPS-stimulated mRNA levels of TNF-α and IL-1β as well as TNF-α-stimulated activation of NF-κB-DNA binding in MNC cells, compared to the placebo group [67]. These findings clearly suggest the beneficial effects of zinc as an anti-inflammatory and pro-antioxidant mediator in SCD patients.

### 7.5. Diabetes Mellitus

Diabetes mellitus (DM), in particular type II, is a major metabolic disease, which is widely prevalent worldwide, especially in developed countries. Accumulating evidence from experimental and clinical studies indicates that oxidative stress plays a very important role in the pathogenesis of both types of DM [172]. It is known that significant amounts of free radicals are generated in diabetes by glucose oxidation and non-enzymatic glycation of proteins, resulting in oxidative degradation of glycated proteins. Such aberration of free radical production and its decreased detoxification by anti-oxidant defense mechanisms in the body can cause cellular and molecular damage, including the development of insulin resistance. These consequences of oxidative stress eventually enhance the development of complications of DM. It has been documented that in DM, especially type II, there are decreased levels of zinc in the body [173,174,175,176]. More evidence shows that zinc deficiency is involved in the development and progression of diabetes and that zinc supplementation improves the status of DM, as previously reviewed [176]. Therefore, zinc supplementation might have beneficial effects in DM via several molecular mechanisms including the suppression of ROS generation.

The earlier clinical trial in the patients with DM type I demonstrated that after three months of follow-up, zinc supplementation (30 mg zinc as gluconate daily) decreased lipid peroxidation as measured by blood thiobarbituric acid (a lipid peroxidation biomarker) and increased antioxidant power as measured by blood and erythrocyte glutathione peroxidase [177]. Another earlier clinical trial in patients with DM type II demonstrated that zinc supplementation (30 mg zinc as gluconate daily for 3 months) decreased the blood level of lipid peroxidation as measured by thiobarbituric acid reactive substances (TBARS) compared to the placebo group of the patients [178]. A similar result was found in 56 of the patients with DM type II who received 6 months of zinc supplementation [53].

One meta-analysis study of zinc supplementation on both types of DM has provided supportive evidence showing that zinc supplementation decreases lipid peroxidation and promote glycemic control in DM patients [179]. Another recent more comprehensive meta-analysis study including 111 original research articles of in vitro, in vivo, and clinical trials showed that zinc has an important role in antioxidant defense system against lipid peroxidation, β-cell function, insulin action, glucose homeostasis, and pathogenesis of diabetes and its complications [180]. A more recent clinical trial indicates that six months of 30 mg zinc supplementation can improve glucose metabolism for example in terms of fasting blood glucose, β-cell function, and insulin sensitivity in pre-diabetic subjects [181]. These findings clearly suggest beneficial effects of zinc through several molecular mechanisms including its role in anti-oxidant defense system in DM patients. However, more clinical trials of zinc supplementation in a large sample sizes of DM patients will be required to provide more conclusive evidence for the improvement of DM status.

### 7.6. Alcohol-Related Liver Disease

Alcohol-related liver disease (ALD) is one of the major causes of chronic liver disease worldwide, due to chronic and high consumption of alcohol or alcohol-containing beverages. This leads to fibrosis, the generation and proliferation of smooth muscle α-actin-positive myofibroblasts of periportal and perisinusoidal areas that results in hepatic cirrhosis [182,183].

Aberrant production and accumulation of ROS or oxidative stress molecules and inflammation have been documented to be associated with the pathogenesis of alcohol-related liver disease [93,183,184]. It is known that acetaldehyde, one major intermediate alcohol metabolite in the body, is a highly reactive oxygen species and is highly toxic to several types of cells such as hepatocytes and cardiocytes. Acetaldehyde can deplete endogenous antioxidant compounds such as glutathione and increase lipid peroxidation, DNA oxidation, and protein oxidation as well as mitochondrial damage, resulting in oxidative stress. Alcohol-derived ROS can also directly trigger the inflammatory response via the activation of oxidant-sensitive NF-κB, which up-regulates the expression and production of inflammatory cytokines/molecules such as TNF-α, IL-6, and other cytokines [93,182,185,186]. Thus, alcohol-induced oxidative stress and the consequent inflammatory response can lead to hepatocyte damage.

It has been documented that zinc deficiency is associated with ALD since alcohol consumption increases zinc excretion in the urine and decreases zinc absorption from the intestine [187,188]. Zinc deficiency enhances the development and progression of ALD. As described above, zinc is a potent pro-antioxidant and anti-inflammatory agent. Therefore, zinc supplementation would have beneficial effects on ALD [121,189]. Animal studies show that zinc supplementation can prevent chronic alcohol-induced liver damage in mice through the inhibition of oxidative stress [190]. One in vitro study indicated that 30 μM of zinc treatment attenuated alcohol-induced cell damage in rat hepatic stellate cells by inhibition of mitogen activated protein kinase (MAPK), stress-activated protein kinase (JNK), NF-κB, transforming growth factor (TGF-β), and tumor necrosis factor (TNF-α), and ROS production [191]. Another animal study revealed that the protective role of zinc in ALD might contribute to the activation of the HNF-4α and PPAR signaling pathways by zinc [118]. Therefore, zinc has a protective role against alcohol toxicity-caused liver cell damage by the inhibition of ROS production and accumulation through the regulation of several molecular signaling pathways.

## 8. Conclusions and Perspectives

In this article, we have discussed the role of zinc as an anti-inflammatory and anti-oxidative stress agent or mediator in human health and diseases. In summary, the evidence shows that zinc is essential for normal physiological function in humans for maintaining immunity and function as an pro-antioxidant and anti-inflammatory mediator via the role of its co-factor. Zinc is involved in regulation of several molecular signaling pathways of zinc associated proteins/zinc finger proteins such as NF-κB, A20, PPAR, TTP, HNF-4α, Nrf2, KRAB, and MT/MTF-1. Zinc supplementation may be useful for the improvement and/or treatment of human health and diseases in different populations with zinc-deficient conditions due to its physiological and biological functions of immunity, anti-inflammation, and suppression of oxidative stress. Further studies are needed to fully understand the molecular mechanisms of zinc. Well-designed clinical trials are needed in order to fully appreciate the benefits of zinc as an anti-oxidative and anti-inflammatory agent for human health.
